# Two New Indolyl Diketopiperazines, Trypostatins C and D from *Aspergillus penicilliodes* Speg.

**DOI:** 10.1007/s13659-018-0156-z

**Published:** 2018-03-14

**Authors:** Han Zhang, Hong-Tao Zhu, Dong Wang, Chong-Ren Yang, Ying-Jun Zhang

**Affiliations:** 1 State Key Laboratory of Phytochemistry and Plant Resources in West China, Kunming Institute of Botany, Chinese Academy of Sciences, Kunming, 650201 China; 2 University of Chinese Academy of Sciences, Beijing, 100049 China; 3 Yunnan Key Laboratory of Natural Medicinal Chemistry, Kunming Institute of Botany, Chinese Academy of Sciences, Kunming, 650201 China

**Keywords:** *Aspergillus penicilliodes* Speg., Indolyl diketopiperazines, Trypostatins

## Abstract

Twelve indolyl diketopiperazines **1**–**12** were isolated from the mycelia culture of *Aspergillus penicilliodes* Speg., a dominant microorganism from the post fermentation process of ripe Pu-er tea. Their structures were elucidated by extensive spectroscopic methods. Among them, trypostatins C (**1**) and D (**2**) featuring with a rare methyl vinyl ketone side chain at C-2 are new compounds, while **3** and **4** were obtained for the first time from nature source. The isolates **3**–**12** did not show obvious cytotoxicities against five human cancer cell lines at a concentration of 40 μM.

**Electronic supplementary material:**

The online version of this article (10.1007/s13659-018-0156-z) contains supplementary material, which is available to authorized users.

## Introduction

Diketopiperazines (DKPs) with a scaffold of 2,5-diketopiperazine formed via the condensation of two amino acid residues. Their conformationally constrained six-membered rings made DKPs an increasing attractive pharmacophore in medicinal chemistry, with a broad pharmacological activity spectrum, e.g., antibacterial [1, 2], antifungal [3, 4], antiviral [5], anticancer [6, 7], immunosuppressive [8], neuro-protection [9], and anti-hyperglycemic [10] activities. To date, DKPs have been commonly found in the marine-derived microorganisms and endophytic fungi. The natural ones with anticancer activities were mainly isolated from *Aspergillus* and *Penicillium* genera [11]. Among which, (−)-phenylahistin (halimide) obtained from *A. ustus* NSC-F038, was a representative DKP with a prenylated chain. Its synthetic analogue, NPI-2358, had been approved for the phase-II clinic trial for the treatment of lymphoma or solid lung cancer as a vascular disrupting agent [12].

*Aspergillus penicilliodes* Speg. was isolated previously from the post-fermentation process of ripe Pu-er tea by our group [13]. In the research of the characteristic components responsible for health improvement of ripe Pu-er tea, the strain KIBA0502 of *A. penicilliodes* was studied chemically for its metabolites. This led to the identification of 12 indolyl diketopiperazines. Trypostatins C (**1**) and D (**2**) featuring with a rare methyl vinyl ketone side chain at C-2 are new compounds, while **3** and **4** were obtained for the first time from nature source. Their structures were determined by extensive spectroscopic analysis and by comparison with literature values. Most of the isolates were tested for their cytotoxicities against five human cancer cell lines.

## Results and Discussion

**Fig. 1:**
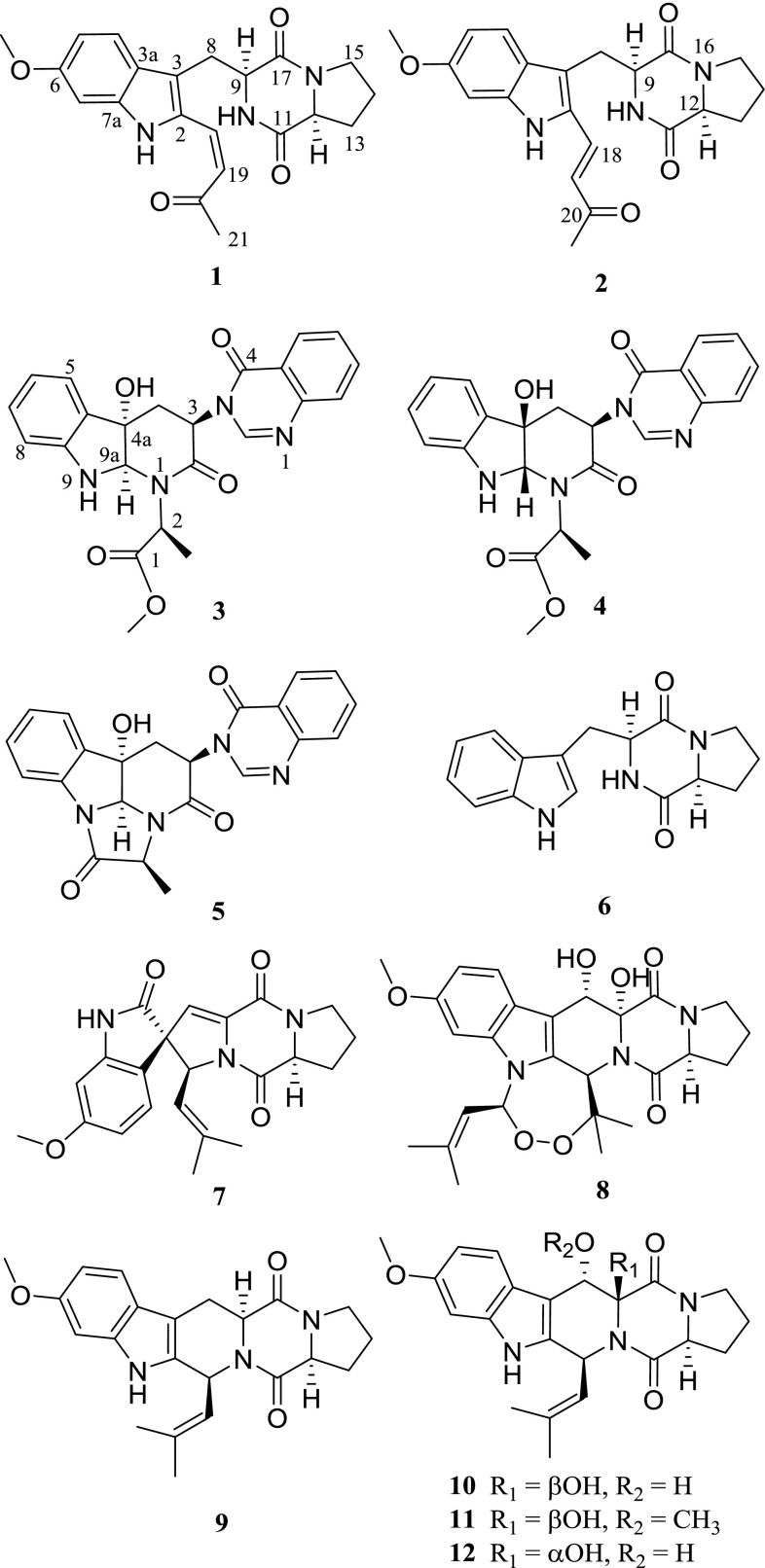
Structures of compounds **1**–**12** from *Aspergillus penicilliodes*

The ethyl acetate extract of the mycelia culture of *A. penicilliodes* (strain KIBA0502) isolated previously from the fermentative stacks of Pu-er tea was applied to repeated column chromatography over silica gel and Sephadex LH-20, followed with semi-preparative HPLC, to afford 12 indolyl diketopiperazines (**1**–**12**) (Fig. 1). Two of them, **1** and **2**, are new compounds.

**Fig. 2:**
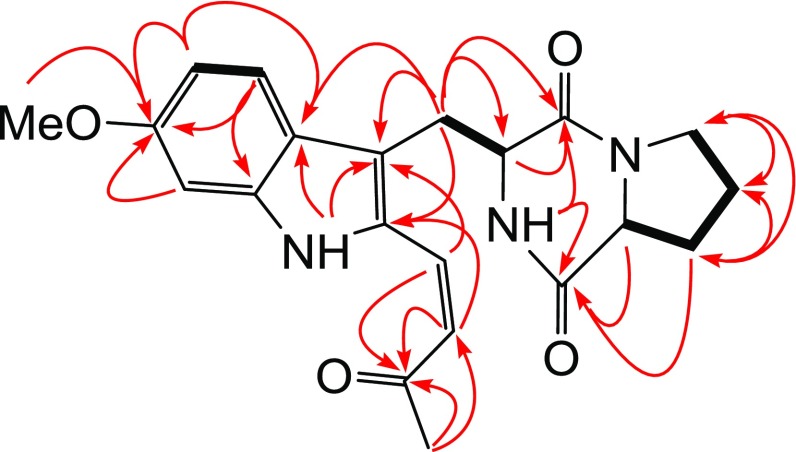
Key ^1^H–^1^H COSY (black bold line) and HMBC (red arrow line from H to C) correlations of compound **1**

Tryprostatin C (**1**) was obtained as a yellow amorphous powder. Its molecular formula was determined to be C_21_H_23_N_3_O_4_ by the HRESIMS (*m/z* 404.1587 [M+Na]^+^). The ^13^C NMR and DEPT spectra of **1** displayed the occurrence of 21 carbon signals assignable to one ketone (*δ*_C_ 199.6), two amide carbonyl (*δ*_C_ 169.4, 165.3), one methoxy (*δ*_C_ 55.7), one methyl (*δ*_C_ 32.0), four methylenes (*δ*_C_ 45.6, 28.5, 25.9, 22.7), two nitrogen-bearing (*δ*_C_ 55.6, 59.3) and two olefinic (*δ*_C_ 129.5, 119.3) methines, and eight aromatic carbons arising from a tri-substituted indolyl unit. The ^1^H NMR spectrum of **1** showed the presence of one *cis*-coupled double bond (*δ*_H_ 6.76, 6.09, each 1H, d, *J* = 12.0 Hz, H-18, H-19), a set of ABX coupled aromatic protons [*δ*_H_ 7.44 (d, *J* = 8.9 Hz, H-4), 6.80 (dd, *J *= 2.2, 8.9 Hz, H-5), 6.89 (d, *J *= 2.2 Hz, H-7)] attributable to a C-6 substituted indolyl unit, two down-field shifted methines [*δ*_H_ 4.23 (dd, *J* = 11.6, 4.2 Hz, H-9), 3.99 (dd, *J* = 7.4, 7.5 Hz, H-12)] assignable to a 2,5-diketopiperazine skeleton, one methoxy (*δ*_H_ 3.81, s) and one methyl (*δ*_H_ 2.33, s). From the well-defined coupling sequences and spatial relationships, three sub-structural fragments could be constructed for **1**, consisting of one 2,3,6-trisubstituted indole, one 2,5-diketopiperazineand one methyl vinyl ketone moieties. These were subsequently reinforced by correlative interpretation of the 2D NMR spectra (Fig. 2), which allowed the unambiguous assignment of all the ^1^H and ^13^C NMR signals. The ^1^H-^1^H COSY correlations constructed obviously three partial structures of –C_(4)_H–C_(5)_H–, –C_(8)_H–C_(9)_H–NH– and –C_(12)_H–C_(13)_H_2_–C_(14)_H_2_–C_(15)_H_2_– in **1**. In the HMBC spectrum, correlation of the methoxy protons at *δ*_H_ 3.81 with C-6 (*δ*_C_ 159.7) indicated its location on C-6. The HMBC correlations of H_2_-8 (*δ*_H_ 3.01, 3.78) with C-2 (*δ*_C_ 131.7), C-3 (*δ*_C_ 117.7), C-3a (*δ*_C_ 122.0), C-9 (*δ*_C_ 55.6), C-17 (*δ*_C_ 165.3) revealed the indole and 2,5-diketopiperazine units were linked with each other through a methylene bridge of C-8. Correlations of *δ*_H_ 2.33 (H_3_-21) with C-20 (*δ*_C_ 199.6) and C-19 (*δ*_C_ 119.3), and both *δ*_H_ 6.76 (H-18) and 6.09 (H-19) with C-20 (*δ*_C_ 199.6) confirmed the presence of methyl vinyl ketone partial structure, which were determined to be connected to the indolyl C-2 by the HMBC correlations of H-19 (*δ*_H_ 6.09) with C-2 (*δ*_C_ 131.7) and H-18 (*δ*_H_ 6.76) with C-3 (*δ*_C_ 117.7). Comparison of the NMR data of **1** with those of tryprostatin A [14] manifested that **1** had a similar indolyl diketopiperazine skeleton, except for the presence of a methyl vinyl ketone side chain in **1**, instead of an isoprenyl group in tryprostatin A. The relative configuration of **1** was established by ROESY experiment. In which, correlation of H-9 with H-12 indicated both protons located at the same side. Based on comparison of its specific rotation with that of tryprostatin A [15], the absolute stereochemistry at C-9 and C-12 in **1** was assigned to be both as *S* configurations, which are the same to those of the other known 2,5-diketopiperazine-type compounds. On the basis of the above evidences, the structure of tryprostatin C (**1**) was deduced as shown in Fig. 1.

Compound **2** obtained as yellow amorphous powder, had a molecular formula of C_21_H_23_N_3_O_4_, as determined by the HRESIMS (*m/z* 404.1581 [M+Na]^+^), which was same to that of **1**. The 1D NMR spectroscopic data were almost the same with those of **1**, suggesting that **2** had the same indole diketopiperazine skeleton. The only different between **2** and **1** was the appearance of a *trans*-coupled double bond [(*δ*_H_ 7.48, 6.36, each 1H, d *J* = 16.0 Hz, H-18, H-19)] in **2**, instead of the *cis* form in **1**. The 2D NMR spectra further revealed that structure of **2** as shown in Fig. 1. The configuration of **2** was deduced to be the same as that of **1**, according to the ROESY correlation of H-9 with H-12, and further confirmed by the specific rotation. Therefore, the absolute configuration of tryprostatin D (**2**) was established to be 9*S*,12*S*.

It is noted, at the room temperature, compound **2** changed rapidly to **1** in a methanol or chloroform solutions. The *cis* conformation of double bond in **1** is obviously more stable than the *trans* form in **2**. This is opposite to the most cases that *trans*-coupled double bonds are more stable than the *cis* ones. It might be the formation of an intramolecular hydrogen bond between 1-NH and the C-20 ketone that caused the C-18/C-19 double bond to be *cis* form in **1**.

Compounds **3**–**12** were 10 known indolyl diketopiperazines. They were identified as methyl(*S*)-{2-((3*R*,4a*S*,9a*R*)-hydroxy-2-oxo-3-(4-oxoquinazolin-3(4*H*)-yl)-2,3,4,4a, 9,9a-hexahydro-1*H*-pyrido[*2,3*-*b*]indol-1-yl)propanoate}(**3**), methyl(*S*)-{2-((3*R*,4a*R*, 9a*S*)-4a-hydroxy-2-oxo-3-(4-oxoquinazolin-3(4*H*)-yl)-2,3,4,4a,9,9a-hexahydro-1*H*- pyrido [2, 3] indol-1-yl)propanoate}(**4**) [16, 17], chaetominine (**5**) [18, 19], brevianamide F (**6**) [20], 6-methoxyspirotryprostatin B (**7**) [21], verruculogen (**8**) [22], fumitremorgin C (**9**) [23], cyclotryprostatin A (**10**), cyclotryprostatin B (**11**) and cyclotryprostatin C (**12**) [24], respectively, by comparison of their spectroscopic data with literature values, and single-crystal X-ray diffraction in the case of **5**. All of the isolates were obtained from *A. penicilliodes* for the first time.

The known compounds **3**–**12** were evaluated for their cytotoxic activities against human myeloid leukemia HL-60, hepatocellular carcinoma SMMC7721, lung cancer A-549, breast cancer MCF-7, and colon cancer SW480 cell lines. However, no compounds showed obvious activity at a concentration of 40 μM.

## Experimental

### General Experimental Procedures

Optical rotations were measured with a P-1020 polarimeter (JASCO, Tokyo, Japan). UV spectra were recorded on a Shimadzu UV2401A ultraviolet–visible spectrophoto-meter. HREIMS spectra were run on a Waters Autospec Premier P776. NMR spectra were measured in CD_3_OD and recorded on a Bruker DRX-600 spectrometer, using TMS as an internal standard. Chemical shifts were reported in units of *δ* (ppm) and coupling constants (*J*) were expressed in Hz. Column chromatography (CC) were carried out silica gel (200–300 mesh, Qingdao Haiyang Chemical Co. Ltd., Qingdao, China), Sephadex LH-20 (25–100 μm, GE Healthcare Bio-Science AB). An Agilent series 1260 (Agilent Technologies) were used for semi-preparative HPLC with an Agilent ZORBAX SB-C18 column (5 μm, 250 mm × 9.4 mm). TLC was performed on pre-coated TLC plates (0.2–0.25 mm thickness, GF254 Silica gel, Qingdao Hailang Chemical Co. Ltd., Qingdao, China) with compounds visualized by spraying with anisaldehyde-sulfuric acid reagent and careful heating.

### Fungal Material

*Aspergillus penicilliodes* Speg. was isolated previously by our group from the fermentative stacks of Pu-er tea produced at Puer specific tea factory in Puer city, Yunnan Province, China, and deposited in China general microbiological culture collection center (CGMCC), Institute of Microbiology, Chinese Academy of Sciences [13]. A loop of spores from a colony growing on potato dextrose agars was inoculated into potato dextrose seed broth (20 g glucose, 200 g potato, 4 g peptone, 0.5 g MgSO_4_, 1.0 g KH_2_PO_4_, and 1000 mL distilled water) for 3 days in the dark at 28 °C, 140 r/min. The seed culture (10 mL) was inoculated into 100 × 500 mL Erlenmeyer flasks, each containing 100 g of wheat immersed in waterand sterilized. Fermentation was conducted under stationary condition for 20 days in the dark at 28 °C.

### Extraction and Isolation

The mycelia culture was extracted three times with CHCl_3_–MeOH (1:1 v/v). After concentrated in vacuo, the resultant extract was suspended into H_2_O and partitioned successively with petroleum ether (4× 3 L), EtOAc (4× 3 L), and *n*-BuOH (4× 3 L). The EtOAc fraction (40.5 g) was subjected to a silica gel column, eluting with CHCl_3_–MeOH (100:0, 98:2, 95:5, 90:10, 85:15, 80:20 v/v) to afford six fractions (Fr. 1-6), which were combined based on TLC analyses. Further separation of Fr.3 (11.0 g) by silica gel CC, using CHCl_3_–MeOH (100, 100:1, 100:2 v/v) as the eluent afforded 12 sub-fractions (subfr.3-1-subfr.3-12). Subfr.3-3 (30 mg) and subfr.3-5 (0.5 g) were separately purified by semi-preparative HPLC (70% MeOH–H_2_O) to give **1** (2.7 mg) and **2** (1.3 mg), and **3** (3.2 mg), **4** (5.0 mg) and **5** (80 mg), respectively. Fr.4 (8.0 g) was applied to Sephadex LH-20 CC, eluting with MeOH–CHCl_3_ (1:1) to afford five sub-fractions (subfr.4-1-subfr.4-5). Subfr.4-3 and Subfr.4-6 were separately subjected to silica gel CC, eluting with a gradient of increasing acetone (0–100%) in petroleum ether to afford subfr. 4-3-1-subfr.4-3-7 and subfr.4-6-1-subfr.4-6-8, respectively. Semi-preparative HPLC (60% aq. MeOH) purification of subfr.4-3-2 (60 mg) and subfr. 4-6-5 (150 mg) afforded **6** (7.0 mg), and **9** (8.2 mg), **10** (23 mg) and **11** (3.0 mg), respectively. Fr.5 (5.5 g) was separated into eightsub-fractions (subfr.5-1-subfr.5-8) by Sephadex LH-20 CC (MeOH-CHCl_3_, 1:1). Subfr.5-4 (62 mg) was purified by semi- preparative HPLC (50% aq. MeOH) to yield **7** (6.4 mg), **8** (2.0 mg) and **12** (5.0 mg).

### Spectroscopic Data

#### Trypostatin C (**1**)

Yellow amorphous powder; [*α*]_D_^20^−24.5 (*c* 0.27, CHCl_3_); UV (MeOH) λ_max_ (log ε): 202 (4.44), 219 (4.13), 398 (3.86) nm; ^1^H and ^13^C NMR data see Table 1; ESI–MS (positive ion mode): *m/z* 404 [M+Na]^+^; HRESIMS (positive ion mode): *m*/*z* 404.1586 [M+Na]^+^, (calcd. for C_20_H_37_NO_6_Na, 404.1586).

**Table 1:** ^1^H and ^13^C NMR spectroscopic data (in CDCl_3_) of **1** and **2**. (*δ* in ppm)

No.	**1**		**2**	
	*δ*_C_	*δ*_H_ (*J* in Hz)	*δ*_C_	*δ*_H_ (*J* in Hz)
1		8.33 (s)		8.28 (s)
2	131.7		131.7	
3	117.7		117.8	
3a	122.0		122.0	
4	120.2	7.44 d (8.9)	120.2	7.44 d (8.9)
5	112.9	6.80 dd (2.2, 8.9)	112.9	6.80 dd (2.2, 8.9)
6	159.7		159.8	
7	94.1	6.89 d (2.2)	94.1	6.89 d (2.2)
7a	137.7		137.7	
8	25.9	3.01 dd (11.2, 15.1) 3.78 dd (4.0, 14.8)	26.0	3.01 dd (11.0, 15.1) 3.78 dd (4.1, 15.2)
9	55.6	4.23 dd (11.6, 4.2)	55.7	4.23 dd (11.6, 4.2)
10		5.41 s		
11	169.4		169.5	
12	59.3	3.99 dd (7.4, 7.5)	59.4	3.99 dd (7.4, 7.5)
13	28.5	1.96 m, 2.27 m	28.5	1.96 m, 2.27 m
14	22.7	1.97 m, 1.84 m	22.8	1.97 m, 1.84 m
15	45.6	3.54 m, 3.59 m	45.7	3.54 m, 3.59 m
16				
17	165.3		165.3	
18	129.5	6.76 d (12.0)	129.6	7.48 d (16.0)
19	119.3	6.09 d (12.0)	119.3	6.36 d (16.0)
20	199.6		199.3	
21	32.0	2.33 s	32.1	2.33 s
OCH_3_	55.7	3.81 s	55.7	3.81 s

#### Trypostatin D (**2**)

Yellow amorphous powder; [α]_D_^20^−61.7 (*c* 0.13, CHCl_3_); UV (MeOH) λ_max_ (log ε): 201 (4.37), 217(4.18), 400 (3.94) nm; ^1^H and ^13^C NMR data see Table 1; ESI–MS (positive ion mode): *m/z* 404 [M+Na]^+^; HRESIMS (positive ion mode): *m*/*z* 404.1581 [M+Na]^+^, (calcd. for C_22_H_39_NO_6_Na, 404.1586).

### Cytotoxicity Assay

Five human cancer cell lines, myeloid leukemia HL-60, hepatocellular carcinoma SMMC7721, lung cancer A-549 cells, breast cancer MCF-7, and colon cancer SW480, were used in the cytotoxic assay. All the cells were cultured in RPMI-1640 or DMEM medium (Hyclone, USA), supplemented with 10% fetal bovine serum (Hyclone, USA).The cytotoxicassay was performed according to the MTS method in 96-well microplates. Briefly, adherent cells (100 μL) was seeded into each well of 96-well cell culture plates and allowed to adhere for 24 h before drug addition, while suspended cells were seeded just before drug addition, each tumor cell line was exposed to the test compound dissolved in DMSO in triplicates for 48 h at 37 °C, with DDP and taxol (Sigma, USA) as positive controls. After the incubation, 20 μL MTS and 100 μL medium was added to each well after removal of 100 μL medium, and the incubation continued for 4 h at 37 °C. The optical density was measured at 492 nm using a Multiskan FC plate reader (Thermo Scientific, USA).

## Conclusion

In this study, 12 indole diketopiperazines **1**–**12** were identified for the first time from *A. penicilliodes*. Among them, **1** and **2** are new compounds, while **3** and **4** are new natural products. So far, the natural diketopiperazines with anti-cancer activities were mainly isolated from *Aspergillus* and *Penicillium* genera, with prenylated side chain as the most representative structural fragment, accounting for the majority of this class of compounds. This is the first time to have obtained indole diketo-piperazines featuring with a methyl vinyl ketone side chain at C-2.

## Electronic supplementary material

Below is the link to the electronic supplementary material.

1D and 2D NMR, ESIMS, HRESIMS, and UV spectra of **1**–**2**, and X-ray molecular structure of **5** are available as Supporting Information (SI). Supplementary material 1 (DOCX 80171 kb)
