# Supplementary material for: Two New Indolyl Diketopiperazines, Trypostatins C and D from Aspergillus penicilliodes Speg
Source: Nat Prod Bioprospect. 2018 Mar 14;8(2):107–11. doi: 10.1007/s13659-018-0156-z (PMC5913049; doi:10.1007/s13659-018-0156-z)
Supplement: Supplementary file 1 — 1D and 2D NMR, ESIMS, HRESIMS, and UV spectra of 1–2, and X-ray molecular structure of 5 are available as Supporting Information (SI). Supplementary material 1 (DOCX 80171 kb) [file 13659_2018_156_MOESM1_ESM.docx]

**Supplementary Information**

**Two new** **indolyldiketopiperazines, trypostatins C and D from** ***Aspergillus penicilliodes* Speg.**

Han Zhang,^a,b^ Hong-Tao Zhu,^a^ Dong Wang,a Chong-RenYang,a Ying-Jun Zhang^a,c,^*

*^a^State Key Laboratory of Phytochemistry and Plant Resources in West China, Kunming Institute of Botany, Chinese Academy of Sciences, Kunming 650201,China.*

*^b^University of Chinese Academy of Sciences, Beijing 100049, China.*

*^c^Yunnan Key Laboratory of Natural Medicinal Chemistry, Kunming Institute of Botany, Chinese Academy of Sciences, Kunming 650201, China.*

* Corresponding author. E-mail: [zhangyj@mail.kib.ac.cn](mailto:zhangyj@mail.kib.ac.cn)

| **No.** | **Contents** | **Page** |
| --- | --- | --- |
| 1 | Figure S1. X-ray molecular structure of chaetominine (**5**) | 3 |
| 2 | Figure S2. ^1^H NMR spectrum of compound **1** | 4 |
| 3 | Figure S3. ^13^C NMR spectrum of compound **1** | 4 |
| 4 | Figure S4. HSQC spectrum of compound **1** | 5 |
| 5 | Figure S5. HMBC spectrum of compound **1** | 5 |
| 6 | Figure S6. ^1^H-^1^H COSY spectrum of compound **1** | 6 |
| 7 | Figure S7. ROESY spectrum of compound **1** | 6 |
| 8 | Figure S8. HRESIMS of compound **1** | 7 |
| 9 | Figure S9. ESIMS of compound **1** | 8 |
| 10 | Figure S10. UV spectrum of compound **1** | 9 |
| 11 | Figure S11. OR spectrum of compound **1** | 10 |
| 12 | Figure S12. ^1^H NMR spectrum of compound **2** | 11 |
| 13 | Figure S13. ^13^C NMR spectrum of compound **2** | 11 |
| 14 | Figure S14. HSQC spectrum of compound **2** | 12 |
| 15 | Figure S15. HMBC spectrum of compound **2** | 12 |
| 16 | Figure S16. ^1^H-^1^H COSY spectrum of compound **2** | 13 |
| 17 | Figure S17. ROESY spectrum of compound **2** | 13 |
| 18 | Figure S18. HRESIMS of compound **2** | 14 |
| 19 | Figure S19. ESIMS of compound **2** | 15 |
| 20 | Figure S20. UV spectrum of compound **2** | 16 |
| 21 | Figure S21. OR spectrum of compound **2** | 17 |

**Figure 1**. X-ray molecular structure of chaetominine (**5**)

**Figure S2**. ^1^H NMR spectrum of compound **1** (600 MHz, CDCl_3_)

**Figure S3**. ^13^C NMR spectrum of compound **1** (600 MHz, CDCl_3_)

**Figure S4**. HSQC spectrum of compound **1** (600 MHz, CDCl_3_)

**Figure S5**. HMBC spectrum of compound **1** (600 MHz, CDCl_3_)

**Figure S6**. ^1^H-^1^H COSY spectrum of compound **1** (600 MHz, CDCl_3_)

**Figure S7**. ROESY spectrum of compound **1** (600 MHz, CDCl_3_)

**Figure S8**. HRESIMS spectrum of compound **1**


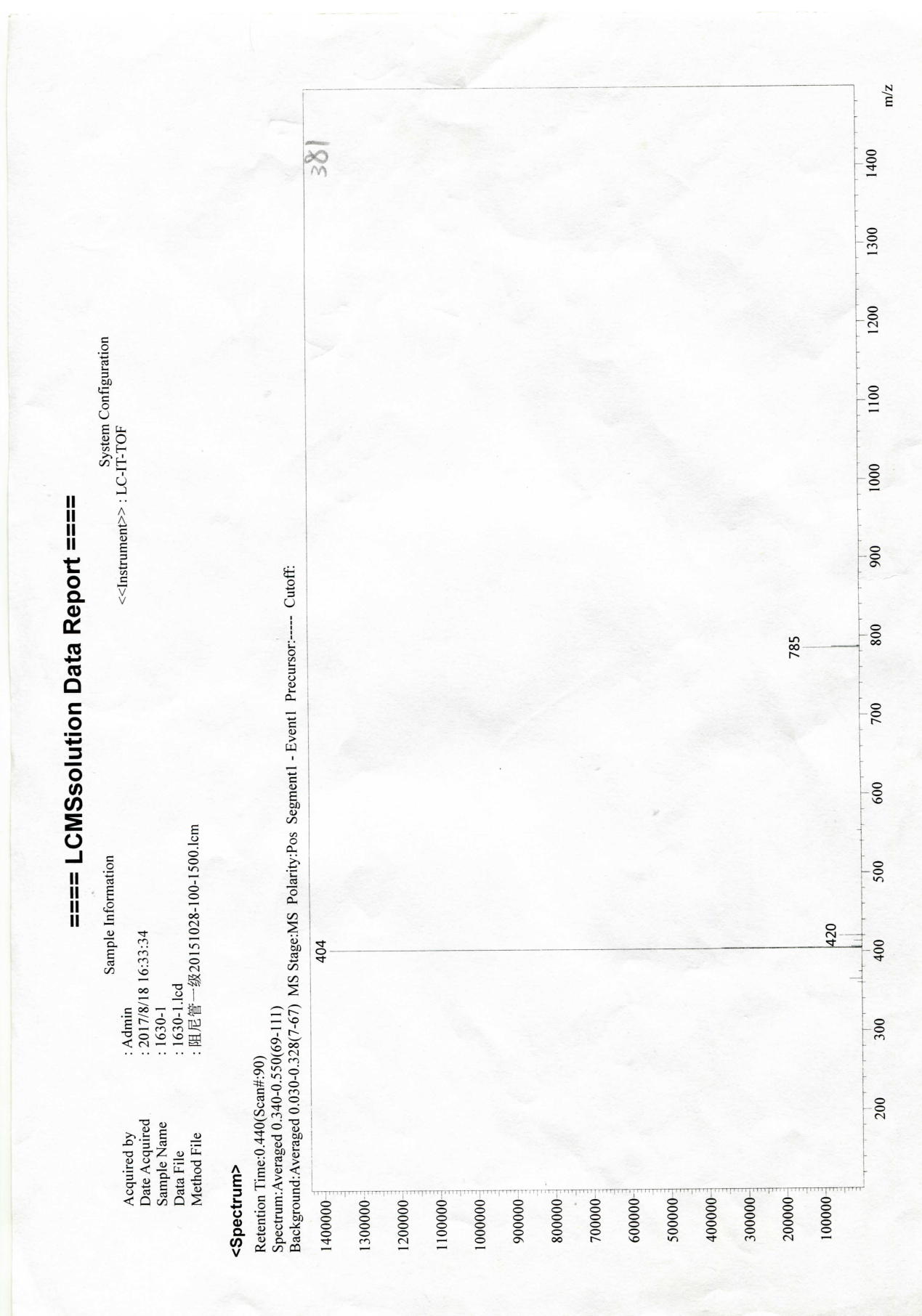


**Figure S9**. ESIMS spectrum of compound **1**


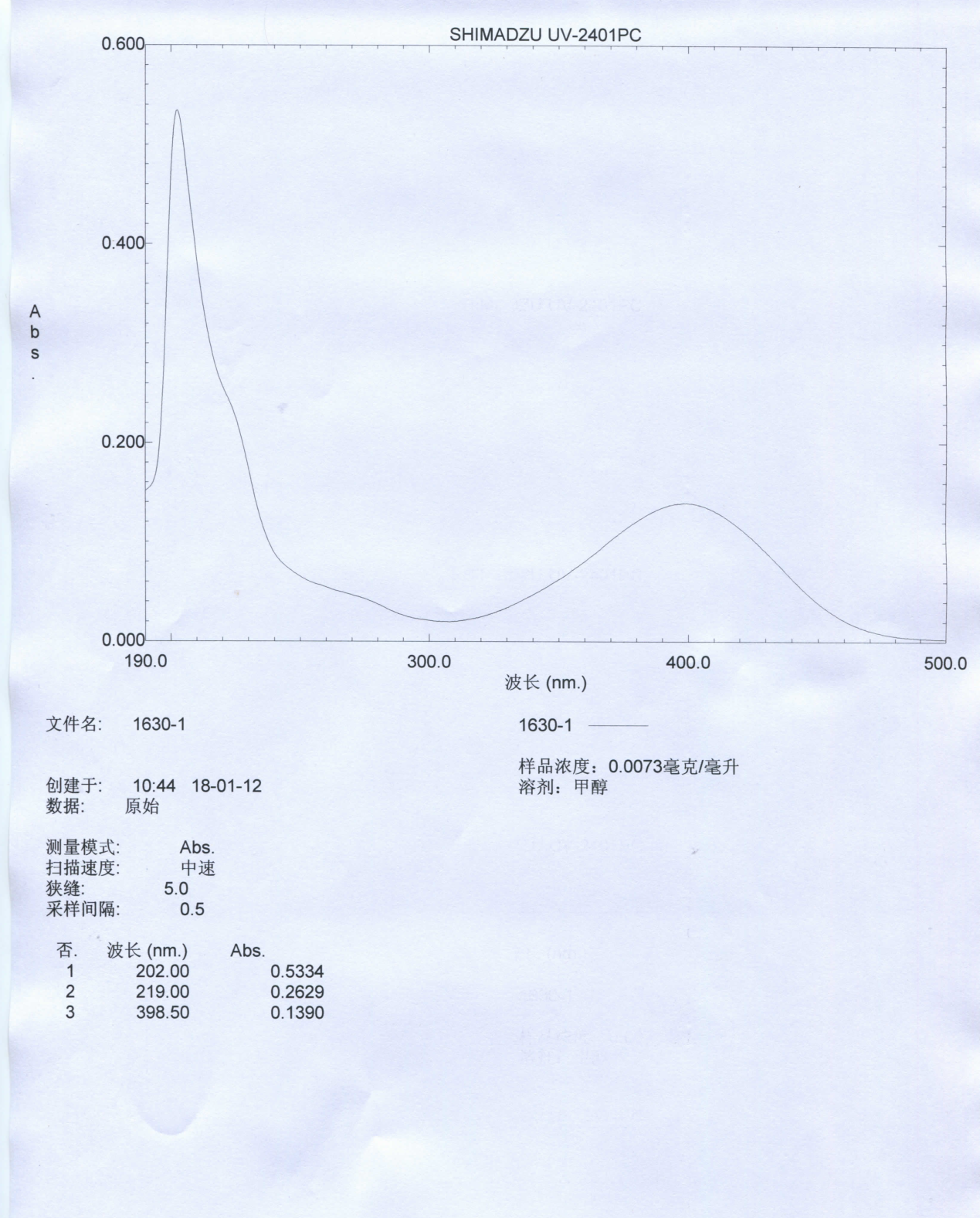


**Figure S10**. UV spectrum of compound **1**


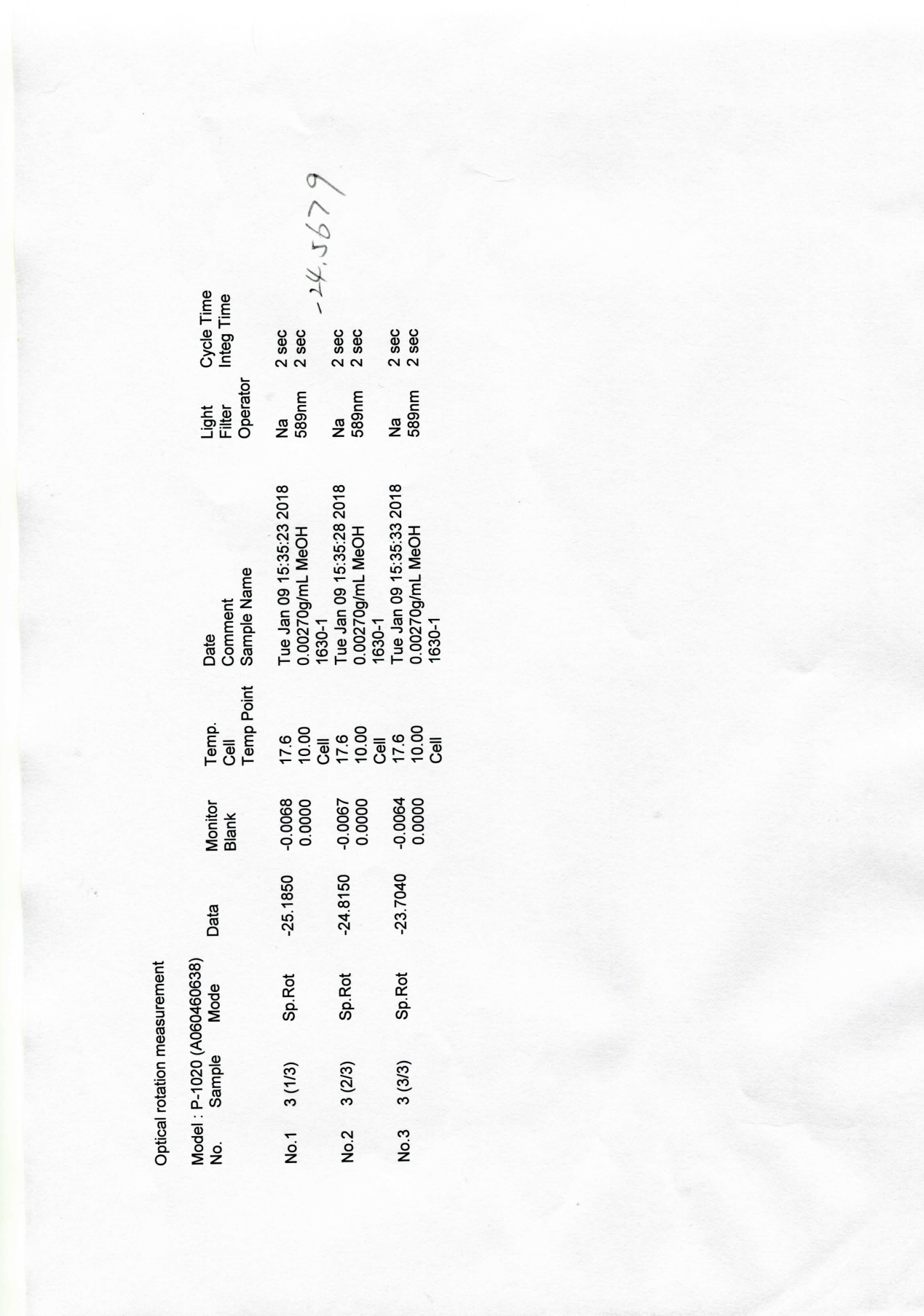


**Figure S11**. OR spectrum of compound **1**

**Figure S12**. ^1^H NMR spectrum of compound **2** (600 MHz, CDCl_3_)

**Figure S13**. ^13^C NMR spectrum of compound **2** (600 MHz, CDCl_3_)

**Figure S14**. HSQC spectrum of compound **2** (600 MHz, CDCl_3_)

**Figure S15**. HMBC spectrum of compound **2** (600 MHz, CDCl_3_)

**Figure S16**. ^1^H-^1^H COSY spectrum of compound **2** (600 MHz, CDCl_3_)

**Figure S17**. ROESY spectrum of compound **2** (600 MHz, CDCl_3_)

**Figure S18**. HRESIMS spectrum of compound **2**


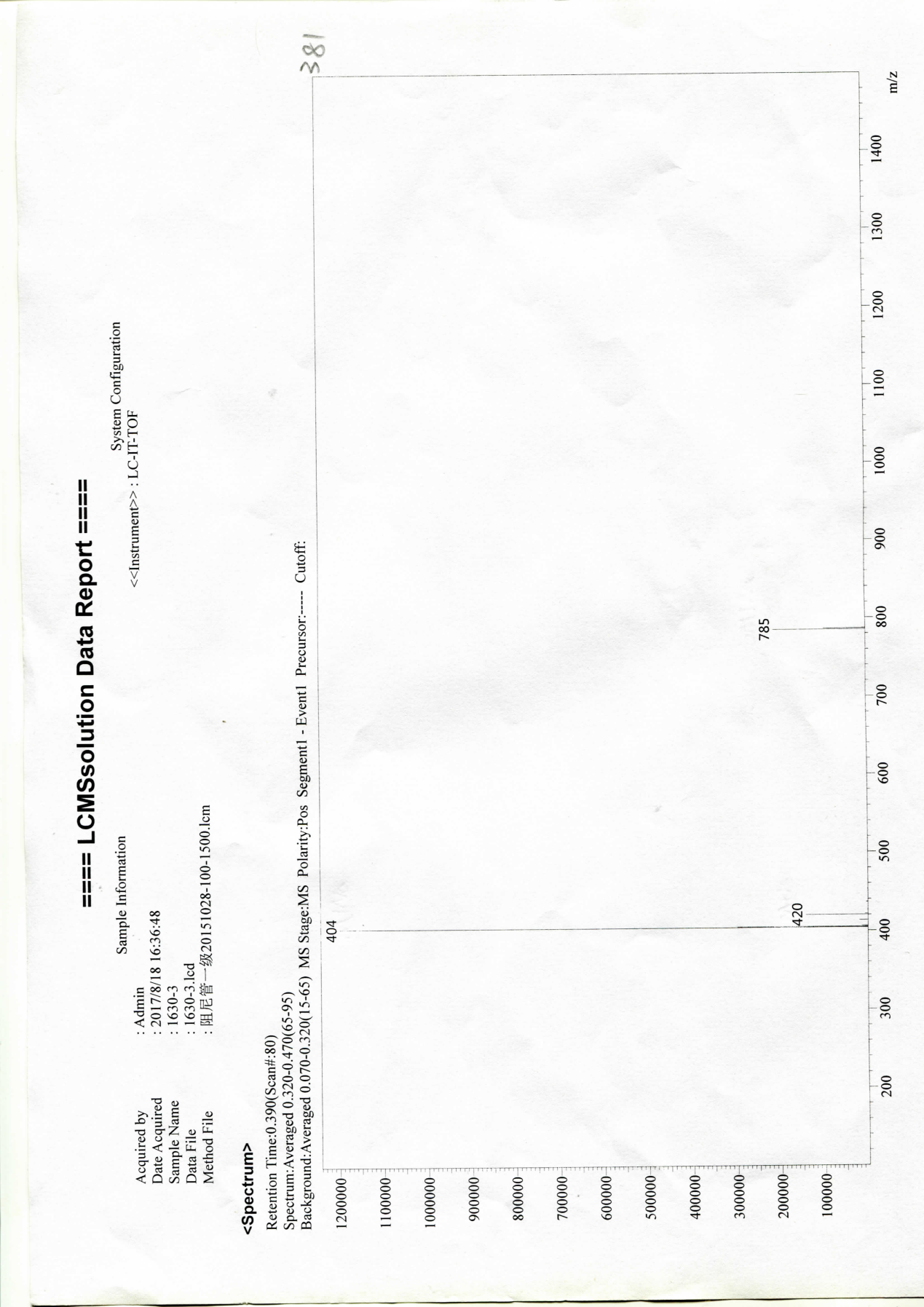


**Figure S19**. ESIMS spectrum of compound **2**


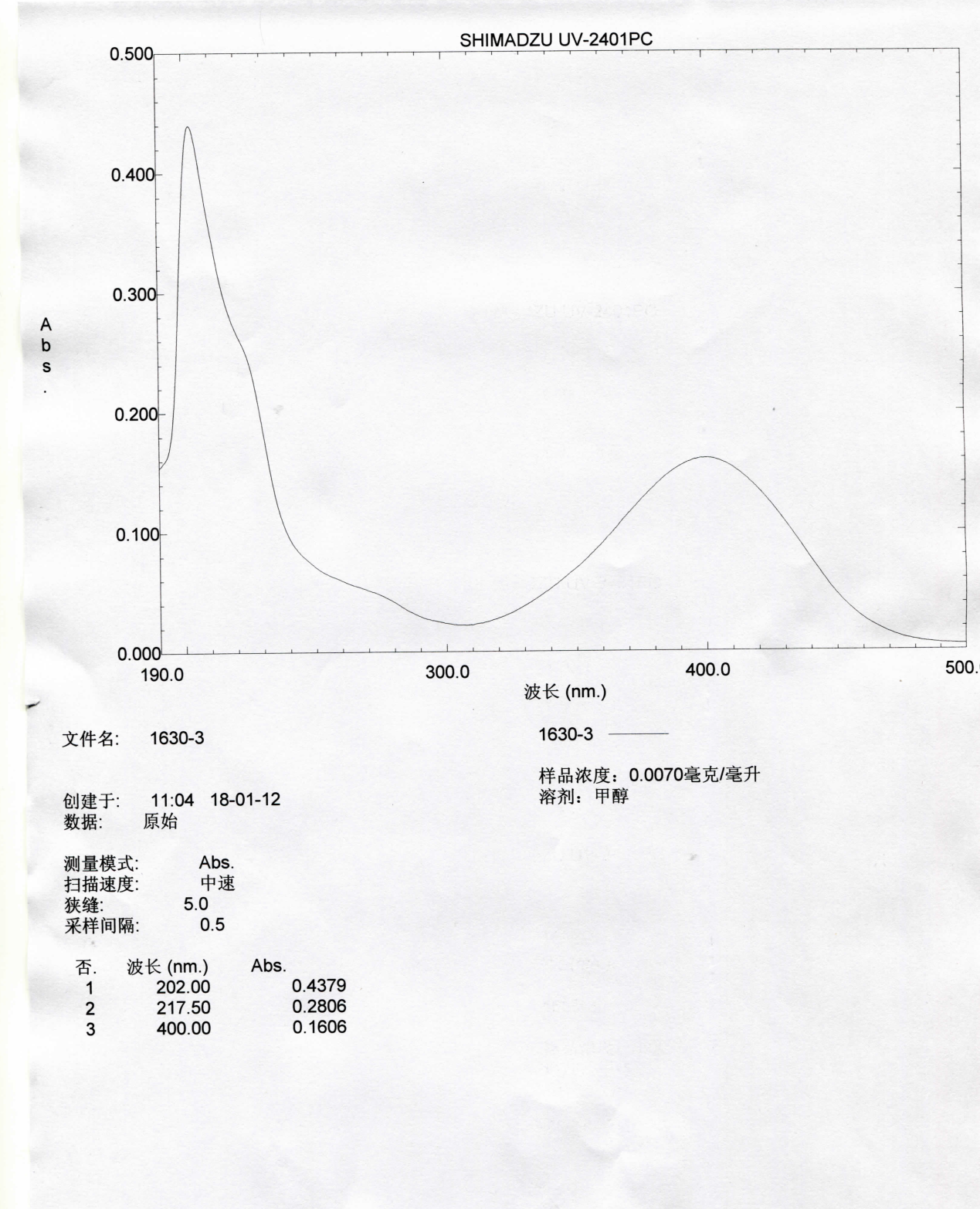


**Figure S20**. UV spectrum of compound **2**

**
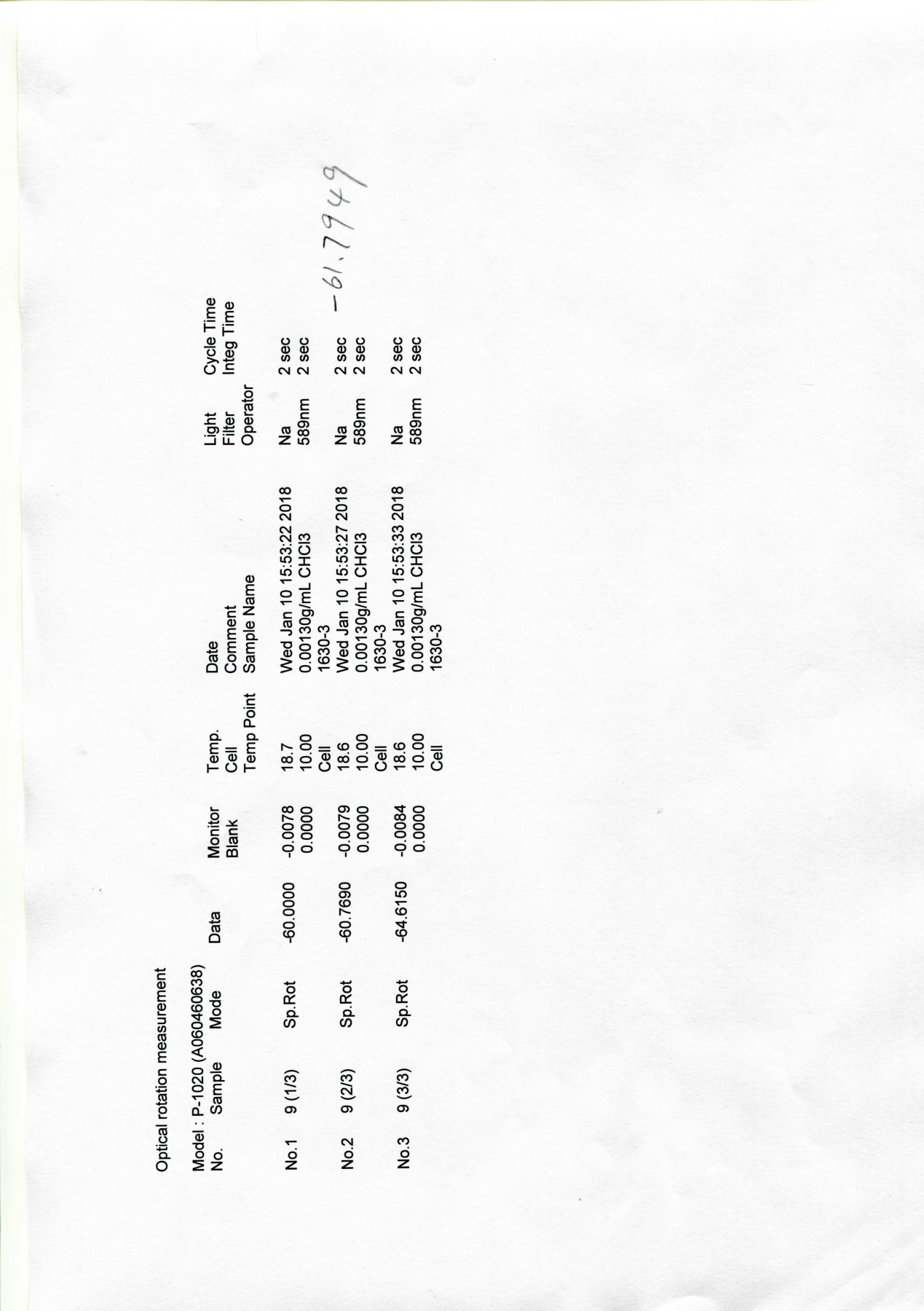
**

**Figure S21**. OR spectrum of compound **2**
